# Hypertensive Retinopathy among Hypertensive Patients in a Tertiary Care Centre: A Descriptive Cross-sectional Study

**DOI:** 10.31729/jnma.8761

**Published:** 2024-09-30

**Authors:** Anjila Basnet, Nilshan Rai, Shambhu Kumar Sahani, Anil Pathak, Bishal Nepali

**Affiliations:** 1 Department of Ophthalmology, KIST Medical College and Teaching Hospital, Imadol, Lalitpur, Nepal; 2 KIST Medical College and Teaching Hospital, Imadol, Lalitpur, Nepal

**Keywords:** *blood pressure*, *hypertension*, *hypertensive retinopathy*, *prevalence*

## Abstract

**Introduction:**

Hypertension manifests in the eyes as retinopathy, choroidopathy, and optic neuropathy of which retinopathy can be used as a predictor for systemic morbidities and mortalities. The study aims to determine the prevalence of hypertensive retinopathy among hypertensive patients visiting the outpatient Department of Ophthalmology in a tertiary care center.

**Methods:**

A descriptive cross-sectional study was conducted among hypertensive patients from 4^th^ April to 19^th^ November 2023 after obtaining ethical approval from the Institutional Review Committee (Reference number: 2079/80/96). A convenience sampling method was used. The point estimate was calculated at a 95% Confidence Interval.

**Results:**

Among 161 hypertensive patients, hypertensive retinopathy was present in 70 (43.48%) (35.82-51.14, 95% Confidence Interval). Grade I hypertensive retinopathy accounted for 35 (50%) followed by Grade II Hypertensive Retinopathy in 17 (24.29%), Grade III Hypertensive Retinopathy in 14 (20%) and Grade IV Hypertensive Retinopathy in 4 (5.71%) in the study participants.

**Conclusions:**

The prevalence of hypertensive retinopathy was found to be higher than other studies done in similar settings.

## INTRODUCTION

Hypertension is one of three leading cause of mortality globally.^1^ Hypertension can affect the eyes by leading to retinopathy, choroidopathy, optic neuropathy, and increasing the risk of retinal artery and vein occlusions, as well as non-arteritic anterior ischemic optic neuropathy, all of which threaten vision.^2^ Retinal blood vessels lack sympathetic nerve supply and have a blood-retinal barrier, so when blood pressure rises, it directly affects these vessels, leading to initial constriction. If the pressure continues to rise, it can cause damage to the muscle layer and endothelium.^3^ Prolonged uncontrolled systemic hypertension may result in visual impairment and blindness.^4^ The retinal microcirculation, visible through noninvasive imaging, provides opportunity to study the link between systemic microvascular disease from hypertension and its connection to cardiovascular conditions.^5^

Hypertensive retinopathy (HR) can be considered for risk stratification to other target organ involvement in a clinical setting. This study aimed to find out the prevalence of hypertensive retinopathy among patients with hypertension in a tertiary care center.

## METHODS

A descriptive cross-sectional study was conducted in the outpatient Department of Ophthalmology at KIST Medical College and Teaching Hospital from 4^th^ April to 19^th^ November 2023. Ethical approval was taken from the Institutional Review Committee (Reference number: 2079/80/96). All the hypertensive patients of both genders above 15 years of age were included in the study. Patients with life-threatening conditions requiring life support and those with known co-morbidities like diabetes mellitus and leukemia that may overlap the pathophysiology of hypertensive retinopathy or any ocular disease like corneal or lens opacities that cause media haze resulting in difficulty in fundus assessment were excluded from the study. A convenience sampling method was used. The sample size was calculated by using the following formula:


$$\begin{array}{ll} \text{n} & ={\text{Z}}^{2}\times \frac{\text{p}\times \text{q}}{{\text{e}}^{\text{2}}}\\ & =1.96^{2}\times \frac{0.107\times 0.893}{0.05^{2}}\\ & =146 \end{array}$$

Where,

n = minimum required sample size
Z = 1.96 at 95% Confidence Interval (CI)
p = prevalence taken from a previous study, 10.7%^6^
q = 1-p
e = margin of error, 5%

The calculated sample size was 146.

A 10% non-response rate was added; n = n + 10% of n = 161

A total of 161 patients were included in the study.

A comprehensive medical history was obtained from all patients, including demographic information, known duration of hypertension, family history of hypertension, history of alcohol intake, smoking history, and comorbidities such as stroke and chronic kidney disease. After explaining the purpose of the study and the confidentiality of data collection, informed consent was obtained from each participant. The study participants were evaluated in detail in the following sequences: visual acuity measurement of each eye separately (unaided and visual acuity with glasses) with internally illuminated Snellen's chart, near vision test, confrontation test, ocular motility assessment, color vision with Ishihara test, cover tests, and refraction using a heine beta 200 retinoscope. Detailed anterior segment evaluation was done with slit lamp biomicroscopy (Shin-Nippon/Aurolab). The posterior segment evaluation was done with direct and indirect ophthalmoscopes (Heine/Welch Allyn/Volk 90D Aspheric lens/Neitz). Tropicamide 1% eye drop was used to dilate the pupils for fundus evaluation. All four quadrants of the retina-superior, inferior, nasal, and temporal were examined in detail. The macula and foveal region were given special attention during the fundus examination. An ophthalmologist examined all the patients and appropriate intervention was taken. The significant findings from the fundus were documented and hypertensive retinopathy was graded as grade I as mild generalized constriction of retinal arterioles; grade II as definite focal narrowing of retinal arterioles plus and arteriovenous nicking; grade III as signs of grade II plus retinal hard exudates, flame-shaped retinal hemorrhages, and cotton -wool spots; and grade IV as severe grade III hypertensive retinopathy plus papilloedema or retinal edema according to the 'Keith-Wagener-Barker' classification.^6^ Blood pressure was measured manually using a sphygmomanometer and stethoscope. Normal blood pressure was defined as systolic blood pressure less than 130 mm Hg and diastolic blood pressure less than 85mm Hg. Mild high blood pressure was defined as systolic blood pressure between 140-159 mm Hg and diastolic blood pressure between 90-00 mm Hg, moderate high blood pressure as systolic blood pressure ranging from 160-179 mm Hg and diastolic blood pressure between 100-109 mm Hg, and severe high blood pressure as systolic blood pressure exceeding 180 mm Hg and diastolic blood pressure over 110 mmHg.^7^

Data were entered in Microsoft Excel 2013 and analyzed by using IBM SPSS statistics version 16.0. The point estimate was calculated at a 95% CI.

## RESULTS

Out of 161 hypertensive patients, 70 (43.48%) (35.8251.14, 95% CI) had HR changes in at least one eye. A total of 77 (47.83%) were male and 84 (52.17%) female. Newar ethnic were 54 (33.54%). There were 108 (67.08%) patients with a family history of hypertension and 144 (89.44) patients were on mixed diet (Table 1).

**Table 1:** Socio-demographic variables of the patients (n= 161).

Variables	n (%)
**Gender distribution**	
Male	77 (47.83)
Female	84 (52.17)
**Ethnicity**	
Newar	54 (33.54)
Brahmin	44 (27.33)
Chhetri	34 (21.11)
Mongolian	16 (9.94)
Madhesi	6 (3.73)
Others	7 (4.35)
**Family history of hypertension**	
Yes	108 (67.08)
No	53 (32.92)
**History of smoking**	
Yes	50 (31.06)
No	111 (68.94)
**History of alcohol intake**	
Yes	56 (34.78)
No	105 (65.22)
**Diet**	
Mix (veg+non-veg)	144 (89.44)
Veg	17 (10.56)

Among total male 43 (61.43%) and 27 (38.57%) female had HR (Table 2).

**Table 2:** Socio-demographic variables of the patients with diabetic retinopathy (n= 70).

Variables	n (%)
**Gender distribution**	
Male	43 (61.43)
Female	27 (38.57)
**Ethnicity**	
Newar	19 (27.14)
Brahmin	15 (21.43)
Chhetri	12 (17.14)
Mongolian	12 (17.14)
Madhesi	6 (8.57)
Others	6 (8.57)
**Family history of hypertension**	
Yes	44 (62.86)
No	26 (37.14)
**History of smoking**	
Yes	24 (34.29)
No	46 (65.71)
**History of alcohol intake**	
Yes	33 (47.14)
No	37 (52.86)
**Diet**	
Mix (veg+non-veg)	62 (88.57)
Veg	8 (11.43)

Among all the patients with HR grade I has predominance with 35 (50%) (Table 3).

**Table 3:** Grading of Hypertensive Retinopathy according to the Keith-Wagener-Barker classification (n=70).

Grade	n (%)
I	35 (50)
II	17 (24.29)
III	14 (20)
IV	4 (5.71)

## DISCUSSION

In our study, prevalence of hypertensive retinopathy was 70 (43.48%) involving at least one eye. The prevalence of this study was higher to the study done in Bharatpur district of Nepal in which the hypertensive retinopathy was found to be 10.7%.^6^ A study done in Pokhara, Nepal showed the prevalence of HR as 38.95% which was lower to our study.^8^ Likewise in a study done in tertiary referral eye institute in Nepal showed the prevalence of HR as 83.7% which was higher than the present study.^9^ The high prevalence of HR may be due to late presentation of the patients to the hospital, uncontrolled hypertension, patients not taking anti-hypertensive medication regularly and lack of awareness of hypertension in the society. In contrast to this study, several studies found low prevalence of HR.^10-12^ The low prevalence of HRin those studies may be due to less duration of hypertension and good blood pressure control.

In our study, HR was commonly seen in male compared with female which was similar to other studies.^13-16^ Similar to our study, one study from Pokhara concluded that HR is common among hypertensives and males are more prone to retinopathy than females.^11^ However, in contrast to our study from Tribhuvan University Teaching Hospital found hypertensive retinopathy more in female than in male.^17^ The variation may be explained by differential distribution of risk factors (e.g. genetic predisposition, dietary factors and lack of physical activities).

In the present study, by Keith-Wagener-Barker classification the prevalence of grade I HR was the commonest accounting for 35 (50%) followed by grade II HR in 17(24.29%), Grade III HR in 14(20%) and Grade IV HR in 4(5.7%). Similarly, other studies also found that the maximum number of participants had Grade I HR followed by grade II HR.^5,18^ Likewise, one study found that there is the risk of HR in the presence of microalbuminuria. They showed that patients with microalbuminuria were more likely to have HR than patients without it. Grade III and IV hypertensive retinopathy were more common in patients with microalbuminuria than in those without it.^19^ Meanwhile one study concluded that that mild HR was positively associated with cardiovascular disease and stroke risk in the urban Japanese population. Specially, generalized arteriolar narrowing and enhanced arteriolar wall reflex were positively associated with cardiovascular disease risk.^20^ Patient with HR should be made aware regarding possible end-organ damage and should be counseled regarding blood pressure control, compliance with treatment and routine follow up. This could help to avoid vision threatening and serious life -threatening consequences and may also reduce the financial burden. Smoking and alcohol consumption has been reported as a risk factors in many studies.^21-23^ However, in our study, HR was lower among those who smoke. This dissimilarity could be related to differences in frequency of smoking and other concurrent comorbid conditions.^24^

Mild HR signs, such as generalized and focal retinal arteriolar narrowing and arteriovenous nicking, are weakly associated with systemic vascular disease. Moderate HR signs, such as isolated microaneurysms, hemorrhages and cotton-wool spots, are strongly associated with subclinical cerebrovascular disease and predict incident clinical stroke, congestive heart failure and cardiovascular mortality, independent of blood pressure and other traditional risk factors.^4^ There have been several experimental studies and clinical reports of regression of HR signs with control of blood pressure. With adequate hypertension treatment, resolution of HR may occur over a period of six months. Thus, follow-up of patients for up to a year after diagnosis may be needed.^5^ In this study, majority of the participants 144 (89.44%) were on mixed diet (vegetarian and non-vegetarian). Similar to this study, a substantial body of evidence from animal studies, epidemiologic studies, meta-analysis, and randomized controlled trials has been demonstrated that certain dietary patterns and individual dietary elements play a prominent role in the development of hypertension. Changes in diet can lower blood pressure, prevent the development of hypertension, and reduce the risk of hypertension-related complications such as HR. Dietary strategies for the prevention of hypertension include reducing sodium intake, limiting alcohol consumption, increasing potassium intake, and adopting an overall dietary pattern such as Dietary Approaches to Stop Hypertension diet or a Mediterranean diet. In order to reduce the burden of blood pressure-related complications, efforts that focus on environmental and individual behavioral changes that encourage and promote healthier food choice are warranted.^25^

The limitation of this study was the patients with moderate HR signs may benefit from further assessment of cardiovascular risk (e.g., assessment of cholesterol Patients with Hypertension Attending the Department of Ophthalmology in a Tertiary Care Centre: A Descriptive levels) and, if clinically indicated, appropriate risk reduction therapy. (e.g., cholesterol-lowering agents was not assessed. This was a hospital based cross-sectional study conducted in one geographical area only. Hence further large-scale analytical study in different regions of Nepal is required. This study also recommends routine ophthalmological examination of every hypertensive patients and holistic management jointly by physician and ophthalmologist to prevent sight threatening and life-threatening complications. Prompt control of hypertension and regular treatment would be helpful to avoid complications. Increased emphasis should be given for awareness to reduce the preventable blinding sequelae and life-threatening complications due to hypertension.

## CONCLUSIONS

The prevalence of hypertensive retinopathy was found to be higher than other studies done in similar settings. Poorly controlled hypertension affects several system such as cardiovascular, renal, cerebrovascular, and retina. Ophthalmologists and general physicians should work in collaborations to ensure the hypertensive patients are efficiently screened, and timely managed to reduce the risk of ocular and systemic morbidity and mortality due to target-organ-damage.
